# The yield of early-pregnancy homeostasis of model assessment -insulin resistance (HOMA-IR) for predicting gestational diabetes mellitus in different body mass index and age groups

**DOI:** 10.1186/s12884-023-06113-3

**Published:** 2023-11-28

**Authors:** Sima Hashemipour, Mahnaz Zohal, Leila Modarresnia, Sepideh Kolaji, Hamidreza Panahi, Milad Badri, Sarah Mirzaeei Chopani, Sara Esmaeili Kelishomi, Amirabbas Ghasemi, Seyyed Hamidreza Ghafelehbashi

**Affiliations:** 1 Metabolic Diseases Research Center, Research Institute for Prevention of Non-Communicable Diseases, Qazvin University of Medical Sciences, Qazvin, Iran; 2 Medical Microbiology Research Center, Qazvin University of Medical Sciences, Qazvin, Iran

**Keywords:** Gestational Diabetes Mellitus, HOMA-IR, Advanced maternal age, Body mass index

## Abstract

**Background:**

Early prediction of gestational diabetes mellitus(GDM) can be beneficial for lifestyle modifications to prevent GDM. The aim of this study was to investigate the predictive values of Homeostasis of Model Assessment -Insulin Resistance (HOMA-IR) in early pregnancy to predict GDM development in different body mass index (BMI) and age risk categories.

**Materials and methods:**

This study is part of the Qazvin Maternal and Neonatal Metabolic Study (QMNMS) in Iran (2018–2021). In this prospective longitudinal study, pregnant women with a gestational age ≤ 14 weeks were enrolled in the study using convenience sampling method and were followed up until delivery to investigate risk factors for maternal and neonatal complications. Data collection was done using questionnaires. Serum sampling was done at a gestational age ≤ 14 weeks and sera were frozen until the end of study. GDM was diagnosed at 24–28 weeks of pregnancy using 75gr oral glucose tolerance test. Fasting blood glucose and insulin were measured in sera taken during early pregnancy in 583 participants. The Mann-Whitney U test, independent t-test, and Chi-square test were used for comparing variables between groups. The logistic regression analysis was used to examine the independent association of HOMA-IR with GDM development and receiver operating characteristic analysis was used for finding the best cut-off of HOMA-IR for predicting GDM.

**Results:**

GDM was developed in 90 (15.4%) of the participants. The third HOMA-IR tertile was independently associated with 3.2 times higher GDM occurrence (95% CI:1.6–6.2, P = 0.001). Despite the high prevalence of GDM in advanced maternal age (GDM rate = 28.4%), HOMA-IR had no association with GDM occurrence in this high-risk group. In both normal BMI and overweight/obese groups, HOMA-IR was a moderate predictor of GDM development (AUC = 0.638, P = 0.005 and AUC = 0.622, P = 0.008, respectively). However, the best cut-off for predicting GDM was 2.06 (sensitivity 67.5%, specificity 61.1%) in normal BMI and 3.13 (sensitivity 64.6%, specificity61.8%) in overweight/obese BMI.

**Conclusion:**

The present study revealed the necessity of considering the BMI and age risk groups when using the HOMA-IR index to predict GDM. Using lower cut-offs is more accurate for women with a normal BMI. In the advanced maternal age, there is no yield of HOMA-IR for predicting GDM.

## Introduction

Gestational diabetes mellitus (GDM) is a highly common pregnancy complication that involves 1–28% of pregnant women [1, 2] and can lead to different complications for the pregnant woman and baby through pregnancy, delivery, and thereafter [3].

The genetic background, high preconception body mass index (BMI), excessive weight gain during pregnancy, advanced maternal age, and a personal history of GDM are the main risk factors for GDM development. From the pathogenesis point of view, insulin resistance and chronic inflammation are the most studied factors in the pathogenesis of GDM [4].

Even a brief contact of the fetus with abnormal metabolic environment in the uterine can lead to the expression of some fetal genes that regulate insulin secretion and action via the epigenetic mechanism and imply a GDM-induced impaired glucose tolerance and metabolic diseases in the future [5]. Therefore, predicting GDM in early pregnancy and some lifestyle modifications can be beneficial for high-risk pregnant women.

Augmented insulin resistance and inadequate beta-cell response have been defined as the main mechanisms of GDM development [6]. However, there are extensive heterogeneities in the predisposing factors and complications of GDM among different risk groups [7]. One of the most important issues is the mechanisms involved in the GDM of lean women. GDM is not rare in normal-weight women without other obvious risk factors for insulin resistance [8, 9]. In the study by Zhang et al. in 41,845 pregnant women, GDM was found in 7.9% of normal weight women [8]. In the multicenter prospective study by Aydın et al. in Turkey, the prevalence of GDM in normal weight pregnant women was reported to be 11% [9]. The prevalence of GDM in lean women of some ethnicities is even higher; in the study by Furukawa et al. on Japanese women, 36% of pregnant women with GDM were lean [10]. Insulin secretion defects have been reported as the main pathophysiological mechanism of GDM in lean women with GDM [11, 12]. There are limited data on the role of early gestational insulin resistance in GDM development in women with a normal weight.

Advanced maternal age is another known risk factor for GDM development. Generally, advanced maternal age is defined as age ≥ 35 years at the time of delivery [13]. A linear association between GDM risk and maternal age has been reported even after adjusting for parity and pre-pregnancy BMI [14, 15]. Nevertheless, the role of insulin resistance in the pathogenesis of GDM in advanced-aged pregnant women is less clarified.

The incidence of diabetes mellitus increases with aging [16]. Aging is related to higher insulin resistance due to increased visceral fat, reduced lean muscle bulk, and chronic inflammation [17]. Aging is also associated with beta-cell dysfunction and decreased insulin secretion [18]. Despite the high prevalence of GDM in advanced maternal age, there are limited data on the role of insulin resistance in GDM in this high-risk group.

HOMA-IR (Homeostasis Model Assessment of Insulin Resistance) has been used as an index of insulin resistance to predict different aspects of health outcomes in various studies. Higher HOMA-IR values are associated with increased risk of type 2 diabetes, hypertension, and nonfatal cardiovascular adverse events [19]. Regarding GDM, the predictive value of HOMA-IR varied from moderate to strong predictor of this complication in different studies [20–22]. However, the heterogeneity of GDM and the different roles of insulin resistance in different risk groups have been less addressed in most of these studies.

Accordingly, this study aimed to examine the predictive value of HOMA-IR (Homeostasis of Model Assessment Insulin Resistance) as an index of insulin resistance in early pregnancy to predict GDM development in different BMI and age categories.

## Materials and methods

This study is a part of Qazvin Maternal and Neonatal Metabolic Pregnancy Outcome Study (QMNMS). QMNMS is an observational prospective longitudinal study on pregnant women in Qazvin, Iran. Pregnant women who visited the obstetric clinic for prenatal care were recruited from September 2018 to May 2020 and from February 2021 to June 2021. The COVID pandemic was the main cause of the interruption in the study. The inclusion criteria were age ≥ 18 y and gestational age ≤ 14 weeks based on the date of the last menstruation or ultrasonography. The participants with pre-gestational diabetes or undiagnosed overt diabetes on the first prenatal visit were excluded from the study. Sampling was carried out by the convenience method.

The details and objectives of the study were explained to the participants in person. participation was voluntary, and all the participants gave their written informed consent. The data were collected in three prenatal visits; the first antenatal visit at ≤ 14th gestational week, during the 22nd -28th gestational weeks, and during the first 6 weeks postpartum. On the first visit, demographic characteristics, history of chronic diseases, pregnancy history, and lifestyle information were collected using pre-study designed questionnaires. Blood samples were taken after 12 h of fasting, maximally in 1 week after the first visit. All serum samples were frozen at -80 °C, and after completion of the study, fasting blood sugar (FBS) and insulin were measured in one laboratory using the same kits. All participants were screened for GDM using 75gr oral glucose tolerance test at 24–28 gestational week. Normal OGTT was defined as FBS < 92 mg/dL, 1-h glucose < 180 mg/dL, and 2-h glucose < 153 mg/dL. GDM was defined as having at least one measurement higher than these values [23].

FBS and insulin assays were performed via enzymatic and electrochemiluminescence (ECL) methods, respectively, using the Roche/Hitachi Cobas® 6000 immunoassay system and Roche Kits. The inter-assay and intra-assay CV of the insulin assay were 1.2% and 4.5%, respectively.

The HOMA-IR index was calculated as follows [24]:

1 $$HOMA - IR = fasting{\text{ }}blood{\text{ }}sugar{\text{ }}\left( {mg/dl} \right) \times insulin{\text{ }}\left( {mU/{\text{ }}lit} \right)/405$$

This study was approved by the Ethics Committee of Qazvin University of Medical Sciences (IR.QUMS.REC.1399.266).

### Sample size calculation

Considering the prevalence of GDM 10% in general population [25], a relative risk of 2.9 [26], power of 80%, and α = 0.05, the sample sizes in each subgroup was calculated as 156. In the case of advanced maternal age group, regarding the prevalence of about 21% [27] and according to the above considerations, the sample size of advance age group was calculated to be 56 participants.

Because of other objectives of the QMNMS primary study regarding the association of HOMA-IR with other less common outcomes, FBS and insulin levels were measured and HOMA-IR was calculated in a total of 583 serum samples (285 women with BMI < 25 kg/m^2^, 272 women with BMI ≥ 25 kg/m^2^, 481 women aged less than 35 years, and 102 women aged ≥ 35 years).

### Statistical analysis

The statistical analysis was performed in SPSS 24. The Kolmogorov-Smirnov test was used to examine the normality of quantitative data distribution. Quantitative data with and without normal distributions were presented as mean ± SD and median (interquartiles), respectively. Quantitative data with a normal distribution, abnormal distribution, and categorical data between GDM and non-GDM groups were compared using independent t-test, Mann-Whitney U test, and Chi-square test, respectively. Logistic regression analysis was conducted to investigate the independent association of significantly different variables in univariate analysis with GDM occurrence. To assess the association of insulin resistance with GDM occurrence in different age and BMI risk groups, the participants were categorized into age < 35y and age ≥ 35y [13], as well as BMI < 25 kg/m^2^ and BMI ≥ 25 kg/m^2^ [28], and HOMA-IR level and HOMA-IR tertile [29] distributions. These variables were compared between GDM and non-GDM participants in each risk group. The receiver operating characteristic (ROC) analysis was used to find the best cut-off of HOMA-IR for predicting GDM in each group of BMI < 25 kg/m^2^ and BMI ≥ 25 kg/m^2^, separately. P value < 0.05 was considered significant. The Bonferroni test was used for correcting multiple comparisons.

## Results

In total, 583 pregnant women were studies. The baseline characteristics are shown in Table 1. GDM was developed in 90 (15.4%) of the participants. The age of the GDM group was significantly higher than that of the non-GDM group (median and interquartile range in GDM and non-GDM groups: 31.0(8.0)y and 29.0(7.0)y, respectively; P < 0.001). The incidence of GDM was significantly different among the age groups (P < 0.001), and this difference was attributable to women ≥ 35y, in whom the incidence of GDM was 28.4% (P < 0.01 for all comparisons with other age groups).

**Table 1:** Baseline characteristics of participants categorized by GDM development in later months

	GDM (N = 90)	Non-GDM (N = 493)	P
Age (year)	31.0(8.0)	29.0(7.0)	0.001
Age groups			< 0.001*
Group 1 < 25(y)	12 (10.6%)	101(89.4%)	
Group 2 25-29.9(y)	23 (12.9%)	155(87.1%)	
Group 3 30-34.9(y)	26 (13.7%)	164(86.3%)	
Group 4 ≥ 35(y)	29(28.4%)	73(71.6%)	
Gravidity	2.0(2.0)	2.0(1.0)	0.203
Parity			0.549
Nulliparous	38(14.4%)	225 (85.6%)	
Multiparous	52(16.2%)	268(83.8%)	
History of GDM†			< 0.001
Positive	10(41.7%)	14(58.3%)	
Negative	42(14.2%)	254(85.8%)	
BMI before pregnancy	25.6(4.6)	24.6(5.1)	0.018
BMI groups††			0.014
BMI < 25 kg/m^2^	34(11.9%)	251(88.1%)	
BMI ≥ 25 kg/m^2^	53(19.5%)	219(80.5%)	
Weight gain††† (Kg)	9.2 ± 4.6	8.8 ± 4.6	0.506
FBS (mg/dl)	94.0(13.0)	90.0(10.0)	< 0.001
Insulin(mU/L)	13.1(8.3)	9.8(6.9)	< 0.001
HOMA-IR †††	3.1(2.1)	2.2(1.7)	< 0.001
HOMA-IR tertiles			< 0.001**
First tertile	16(8.2%)	179(91.8%)	
Second tertile	26(13.5%)	167(86.5%)	
Third tertile	48(24.6%)	147(75.4%)	

Parametric data are presented by mean ± SD; non-parametric data are presented by median(interquartile); GDM: gestational diabetes mellitus

Using Bonferroni correction for variables with three subgroups, P < 0.017 was set as significant in these comparisons

* Significant differences between age ≥ 35 y with all the other age groups (Group 4 vs. Group 1, p = 0.001, Group 4 vs. Group 2, P = 0.001, and Group 4 vs. Group 3, P = 0.002)

** Significant difference between Tertile 3 and the other two groups (Tertile 3 vs. Tertile 1, P < 0.001, Tertile 3 vs. Tertile 2, P = 0.005)

† Based on the data of 320 multiparous participants

†† Missing BMI data in 26 participants

†††Weight gain until the 24th -28th gestational week

Among 320 multiparous participants, 24 women had positive GDM history in previous pregnancies. GDM occurred in 41.7% of women with GDM history and 14.2% of women without GDM history (P < 0.001).

The incidence of GDM in participants with a BMI of ≥ 25 kg/m^2^ was higher than in normal-BMI ones (19.5% vs. 11.9%, respectively; P = 0.014).

The median (interquartile range) of HOMA-IR in women who eventually developed GDM and the non-GDM groups was 3.1(2.1) and 2.2(1.7), respectively (P < 0.001). GDM was developed in 8.2%,13.5%, and 24.6% of the participants with the first, second, and third tertiles of HOMA-IR, respectively (P < 0.001).

The results of the logistic regression analysis of GDM predictors are presented in Table 2. In Model 1 (the model without entering the HOMA-IR index), the relative risks of GDM occurrence in the groups of age ≥ 35y, being overweight/obese before pregnancy, or having positive GDM history were 2.6(95% CI:1.5–4.5), 1.5(95% CI: 0.9–2.4), and 4.3(95% CI: 1,8-10.3), respectively. After entering the HOMA-IR tertiles into the model (Model 2), age ≥ 35y and positive history of GDM remained significant predictors of GDM [RR = 2.9 (95% CI:1.7-5.0, P < 0.001) and RR = 3.3 (95%CI:1.3-8.0, P = 0.01), respectively].

**Table 2:** Predictors of GDM in logistic regression analysis

	Crude RR(95%CI)		Model 1 RR(95%CI)		Model 2 RR(95%CI)	P
Age ≥ 35y	2.7(1.6–4.5)	< 0.001	2.6(1.5–4.5)	< 0.001	2.9(1.7-5.0)	< 0.001
GDM history	4.3(1.8–9.9)	0.001	4.3 (1.8–10.3)	0.001	3.3(1.3-8.0)	0.01
BMI ≥ 25 kg/m^2^	1.8(1.1–2.8)	0.015	1.5(0.9–2.4)	0.094	1.1(0.6–1.8)	0.724
HOMA –IR tertiles		< 0.001				0.001
1st tertile	Reference				1	
2nd tertile	1.7(0.9–3.4)	0.098			1.6(0.9–3.1)	0.196
3rd tertile	3.7(1.9–6.7)	< 0.001			3.2(1.6–6.2)	0.001

In Model 1, age categories (≥ 35y vs. <35y), GDM history, and BMI categories (≥ 25 kg/m^2^ vs. BMI < 25 kg/m^2^) were entered into the model. In Model 2, HOMA-IR tertiles were added to Model 1

To investigate the association of insulin resistance with GDM development in different risk groups, the level of HOMA-IR and the distribution of HOMA-IR tertiles were compared in age and BMI risk categories (Table 3).

**Table 3:** Association of HOMA-IR with GDM development in age and BMI risk groups

Age subgroups							BMI subgroups					
	**Age < 35y**			**Age ≥ 35y**			**BMI < 25 kg/m** ^**2**^			**BMI ≥ 25 kg/m** ^**2**^		
	GDM	Non-GDM	p	GDM	Non-GDM	p	GDM	Non-GDM	p	GDM	Non-GDM	P
HOMA-IR level	3.2(2.1)	2.2(1.7)	< 0.001	2.2(1.5)	2.3(1.6)	0.873	2.5(1.5)	1.8(1.3)	0.005	3.3(2.2)	2.8(1.6)	0.008
HOMA-IR tertiles			< 0.001			0.750			0.008			0.014
1st tertile N (%)	10 (14.7%)	163 (36.4%)		6 (27.2%)	16 (35.6%)		11 (27.5%)	122 (49.8%)		5 (10.4%)	45 (20.0%)	
2nd tertile N (%)	18 (26.5%)	151 (33.7%)		8 (36.4%)	16 (35.6%)		14 (35.0%)	77 (31.4%)		11 (22.9%)	81 (36.2%)	
3rd tertile N (%)	40 (58.8%)	134 (29.9%)		8 (36.4%)	13 (28.8%)		15 (37.5%)	46 (18.8%)		32 (66.7%)	98 (43.8%)	

The HOMA-IR level is presented by median(interquartile)

Despite the high prevalence of GDM in women aged ≥ 35y (frequency of GDM 28.4%), women who developed GDM in this age group had similar HOMA-IR levels compared with non-GDM one [median(interquartile): 2.2(1.5) and 2.3(1.6), respectively, P = 0.873]. Moreover, the distributions of HOMA-IR tertiles did not show any difference between GDM and non-GDM groups in women aged ≥ 35y. However, in the age category of < 35y, women with GDM had higher levels of HOMA-IR compared to non-GDM ones [3.2(2.1) vs. 2.2(1.7), respectively; P < 0.001] as well as having a higher frequency of the 3rd HOMA-IR tertile of HOMA-IR in the GDM group compared to the non-GDM one (P < 0.001) (Table 3).

Regarding BMI categories, baseline HOMA-IR levels were significantly higher in the GDM group vs. the non-GDM group, either in BMI < 25 kg/m^2^ or BMI ≥ 25 kg/m^2^ groups (P = 0.005 in BMI 25 kg/m^2^ and P = 0.008 in BMI ≥ 25 kg/m^2^). Besides, the distributions of HOMA-IR tertiles differed between GDM and non-GDM groups in both BMI < 25 kg/m^2^ or BMI ≥ 25 kg/m^2^ categories (P = 0.008 and P = 0.014, respectively). Despite the similarity of predictive patterns of baseline HOMA-IR for GDM development in normal and overweight/obese groups, there were some differences regarding levels and distributions of HOMA-IR tertiles in GDM patients in these two BMI groups. In the overweight/obese category, 66.7% of the GDM group had the highest tertile of HOMA-IR, while in GDM patients with BMI < 25 kg/m^2^, the frequency of having the third tertile of HOMA-IR was 37.5% (Table 3).

**Fig. 1:**
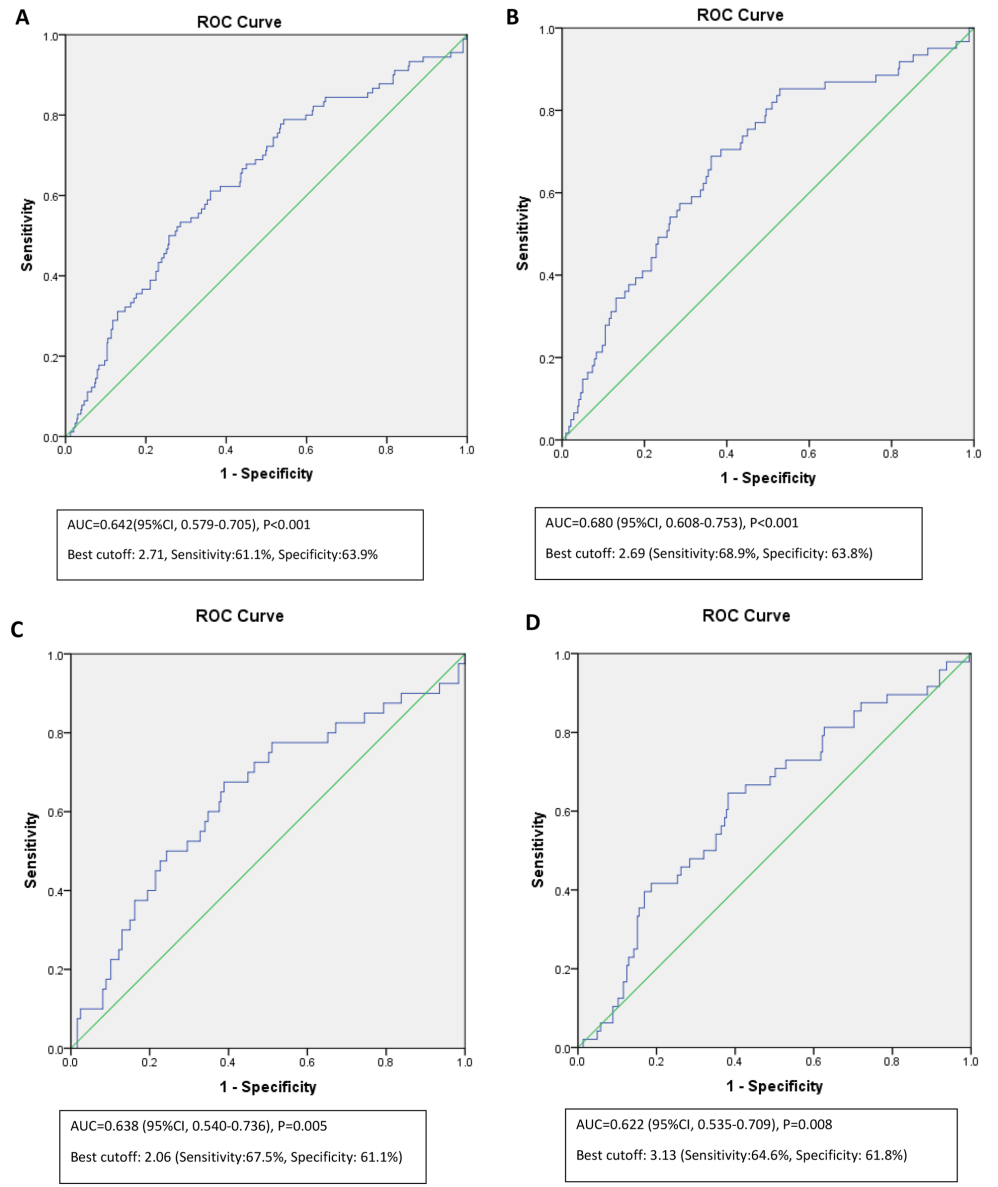
Receiver operating characteristic curve of HOMA-IR for prediction of GDM. **A:** total group, **B:** age<35y, **C:** BMI<25kg/m^2^, **D:** BMI≥25kg/m^2^; ROC: Receiver operating characteristic

Considering the above-mentioned differences of HOMA-IR for predicting GDM development in BMI categories, the ROC curve analysis was performed for total participants, BMI groups, and age < 35y group (Fig. 1). For the total group, the areas under the curve (AUC) was 0.642 (P < 0.001) with the best cutoff of HOMA-IR of 2.71 with sensitivity and specificity of 61.1% and 63.9%, respectively. In the age group of less than 35y, the AUC was 0.680 (P < 0.001) and the best cutoff for predicting GDM was 2.69 (Sensitivity:68.9%, Specificity: 63.8%).

The AUC of normal BMI and overweight/obese groups were nearly similar (AUC = 0.638, P = 0.005 and AUC = 0.622, P = 0.008, respectively). Nevertheless, the best HOMA-IR cut-off for predicting GDM development was 3.13 (sensitivity 64.6%, specificity 61.8%) in the overweight/overweight group, while the best HOMA-IR cut-off was 2.06 (sensitivity 67.5%, specificity 61.1%) in the normal-BMI group.

## Discussion

In this study, the HOMA-IR index in early pregnancy was an independent predictor of GDM development in later months. However, the predictive value of this index was dependent to the baseline risk group. In the very-high-risk group, i.e., women aged 35y or more (with a GDM rate of about one-third of women), HOMA-IR had no predictive value for GDM development. In both normal-BMI and overweight/obese women, the HOMA-IR level was a moderate predictor of GDM; however, the best cut-off of HOMA-IR for predicting GDM was higher in the overweight/obese group.

The predictive value of the HOMA-IR index in early pregnancy for predicting GDM development has been examined in several studies. In some of these studies, the HOMA-IR has been a strong predictor, while in other studies, its predictive value was moderate [20–22]. In the studies by Ozcimen et al. and Alptekin et al., the sensitivity of HOMA-IR for GDM occurrence was reported as 100% and 90%, respectively. In Ozcimen et al.‘s study, the specificity of this index was reported as high as 94%, while in Alptekin et al.‘s study, the specificity was 61%. The best cut-offs for HOMA-IR in these two studies were 2.6 and 2.08, respectively [20, 21]. In other studies, the sensitivity of HOMA-IR for predicting GDM is much lower. In the study by Kirlangiç et al. and our study, the sensitivity of HOMA-IR was found to be 63.6% and 61.1%, respectively [30].

The differences in the reported predictive values of HOMA-IR can be attributed to various baseline risks of participants in different studies and the heterogeneous nature of GDM. Generally, pregnant women with GDM are considered similar; however, substantial heterogeneity has been found between these women [7, 31, 32]. Generally, insulin resistance and inadequate beta-cell response in late pregnancy have been considered as the main pathophysiological mechanisms of GDM [6]. Nevertheless, some differences between lean and obese patients with GDM have been reported in small studies since decades ago. In Cheney et al.‘s study, insulin and glucose responses to meals were compared in lean and obese women with GDM. The fasting and post-meal insulin levels in obese and lean (BMI < normal) women with GDM were higher and lower than those of the control group (normal BMI), respectively. The authors concluded that GDM is a heterogeneous abnormality in which the main mechanisms in lean and obese GDM women are insulin deficiency and insulin resistance, respectively [32].

The impact of BMI on the association between insulin resistance and GDM occurrence has been re-considered recently. In Inoue et al.‘s study, different indexes of insulin secretion and resistance were evaluated in lean pregnant women with GDM (BMI < 18.5 kg/m^2^) and compared to the control group without GDM and a similar BMI. The insulinogenic and composite insulin sensitivity indexes were lower in the GDM group, while no difference in HOMA-IR and HOMA-β was found between GDM and non-GDM groups. The authors concluded that β-cell dysfunction is the main pathophysiologic mechanism of GDM in extremely lean Japanese women [33].

In the study by Furukawa et al., women with GDM were divided into two groups with and without insulin resistance using the HOMA-IR index [10]. Among the GDM women, the frequencies of insulin-resistant and non-insulin-resistant women were 64% and 36%, respectively. The insulin-resistant group had a higher BMI compared to the insulin-sensitive one, and HOMA-IR β (as the insulin secretion index) was lower in the insulin-sensitive group.

The association of HOMA-IR and GDM development in different BMI categories of pregnant women was investigated by Duo et al. [26]. A higher HOMA-IR index was a risk factor for GDM development in all three categories of BMI. However, the best cut-off of HOMA-IR for predicting GDM was different among the groups. The best cut-off of HOMA-IR in the normal-weight group was 1.43, while in the obese group, this index was 2.31. Similarly, in our study, despite the significant association of HOMA-IR with GDM occurrence in both normal-weight and overweight/obese BMI groups, the best cut-off of HOMA-IR for predicting GDM was lower in the normal-BMI group compared with the overweight/obese one.

Based on these considerations, the lower cut-off of HOMA-IR for predicting GDM in the normal-BMI group can be attributed to the combination of two abnormalities of decreased insulin secretion (the main mechanism of GDM in extremely lean women) and increased insulin resistance (the main mechanism in overweight/obese women). Taken together, for a more accurate prediction of GDM, it is reasonable to use different HOMA-IR cut-offs for various BMI categories.

Advanced maternal age is another known risk factor for GDM development [14, 15]. In the systematic review by Li et al., the maternal age of 35–39 years and ≥ 40 years were associated with 3.54 and 4.86 times higher GDM compared with women 20–24 years old [14]. In our study, about one-third of women aged ≥ 35 years developed GDM.

There is limited data about the pathophysiology of GDM in advanced maternal age; to the best of our best knowledge, the role of insulin resistance in GDM development in this age category has not been studied. The prevalence of DM type 2 increases with age [34]. Diabetes in older age is a very heterogonous illness related to various levels of decreased insulin sensitivity and insulin secretion defect [35]. Overall, beta-cell dysfunction plays an essential role in the pathophysiology of age-related type 2 DM. A distinct age-related beta-cell dysfunction has been shown in animal and human studies [36]. The self-renewal of pancreatic beta-cells is the main mechanism for preserving beta-cells. This mechanism is dysregulated and proliferation is arrested in advanced age [37]. In our study, despite the high prevalence of GDM in the advanced-maternal-age group, the HOMA-IR indexes of GDM and non-GDM groups were similar. Regarding the change in beta-cell function with aging and our data, the most probable essential mechanism of GDM in advanced maternal age is insulin secretion defects, and the HOMA-IR index is not a beneficial tool for predicting GDM occurrence in this high-risk group.

Our study has some limitations. In the original study of QMNMS, about 19% of the participants were excluded from the final analysis because of loss to follow-up, missing data, or having pre-gestational diabetes [38]. The second limitation was calculating pre-pregnancy BMI based on pregnant women’s statements and not by objective tools.

The main advantage of our study was examining the predictive values of HOMA-IR for GDM development in different risk groups of maternal age and BMI groups.

## Conclusion

The present study revealed the necessity of considering baseline GDM risk groups when using the HOMA-IR index for GDM prediction. Using lower cut-offs for normal-BMI women is more accurate. In women ≥ 35 years old, there is no yield of HOMA-IR for GDM prediction.

## Data Availability

The datasets used or analyzed during the current study are available from the corresponding author upon reasonable request.
